# Impaired Hearing and Systolic Blood Pressure as Potential Markers of Cerebral Infarction After Eclampsia: A Cross‐Sectional Study

**DOI:** 10.1111/1471-0528.70225

**Published:** 2026-03-24

**Authors:** Lina Bergman, Henrik Imberg, Eduard Langenegger, Ashley Moodley, Richard Pitcher, Stephanie Griffith‐Richards, Karl Bergman, Roxanne Hastie, Stephen Tong, Susan P. Walker, Johan Wikström, Catherine Cluver

**Affiliations:** ^1^ Department of Obstetrics and Gynecology Stellenbosch University Cape Town South Africa; ^2^ Department of Women's and Children's Health Uppsala University Uppsala Sweden; ^3^ Department of Obstetrics and Gynecology, Institute of Clinical Sciences, Sahlgrenska Academy University of Gothenburg Gothenburg Sweden; ^4^ Statistiska Konsultgruppen Sweden Gothenburg Sweden; ^5^ Department of Molecular and Clinical Medicine, Institute of Medicine, Sahlgrenska Academy University of Gothenburg Gothenburg Sweden; ^6^ Division of Radiodiagnosis Stellenbosch University Cape Town South Africa; ^7^ Department of Medicine, Institute of Clinical and Molecular Medicine, Sahlgrenska Academy University of Gothenburg Gothenburg Sweden; ^8^ Division of Cardiology, Faculty of Medicine Stellenbosch University Cape Town South Africa; ^9^ Translational Obstetrics Group, Department of Obstetrics and Gynaecology University of Melbourne Melbourne Victoria Australia; ^10^ Mercy Perinatal Mercy Hospital for Women Heidelberg Victoria Australia; ^11^ Department of Surgical Sciences, Neuroradiology Uppsala University Uppsala Sweden

**Keywords:** cerebral infarcts, clinical markers, eclampsia, magnetic resonance imaging

## Abstract

**Objective:**

Eclampsia is associated with short‐ and long‐term neurological deficits. Identifying which women may be at risk is important. Magnetic resonance imaging shows an incidence of 30%–40% of subclinical cerebral infarcts among women with eclampsia. A simple screening tool would be useful to identify at‐risk women. The objective of this study was to explore clinical markers to identify women at highest risk for silent cerebral infarcts in women who have experienced eclampsia.

**Design:**

This was a prospective observational study with cross‐sectional MRI ascertainment conducted at Tygerberg Hospital, a tertiary referral centre in Cape Town, South Africa. Women were prospectively assessed for symptoms and signs known to be associated with eclampsia. Cerebral infarcts were identified using brain magnetic resonance imaging. Associations between clinical variables and the presence of cerebral infarcts were evaluated using logistic regression, with variables significant at the 20% level considered for inclusion in multivariable analyses using stepwise selection.

**Results:**

A total of 49 women with eclampsia were included in the analysis, of whom 33% (*n* = 16) had cerebral infarcts detected on MRI. Highest systolic blood pressure and impaired hearing prior to the eclamptic seizure were the clinical variables most strongly associated with the presence of silent cerebral infarcts, with an area under the receiver operating characteristic curve of 0.72 (95% CI 0.56–0.88). At a risk threshold of 43%, sensitivity was 60% (95% CI 36%–80%) and specificity was 84% (95% CI 67%–93%).

**Conclusions:**

Higher systolic blood pressure and impaired hearing were the clinical features most strongly associated with silent cerebral infarcts in women with eclampsia. These findings highlight potentially useful clinical markers that, following external validation, may support triage decisions regarding neuroimaging and neurological follow up.

## 1 Introduction

Eclampsia is a severe complication of preeclampsia and presents with generalised tonic–clonic seizures. It is often associated with severe hypertension and other maternal end organ injury. Despite being relatively rare in high‐income countries, it remains a common cause of direct maternal death worldwide [1, 2, 3].

Historically, the effects of eclampsia were believed to be completely reversible, not causing persistent neurological sequelae [4]. However, recent data have shown that women who experience eclampsia are at increased risk of long‐term complications, including increased risk of cognitive decline [5, 6, 7, 8], migraine [9], epilepsy [9, 10], white matter lesions [11], stroke [12] and dementia [13].

Studies using Computed tomography (CT) and/or Magnetic Resonance Imaging (MRI) for assessment of oedema and infarcts in eclampsia have shown varying incidences (between 0% and 100%) of vasogenic cerebral oedema and a large variation in incidence of cerebral infarcts in women with preeclampsia and eclampsia [14, 15, 16, 17, 18, 19, 20, 21, 22, 23]. For women with eclampsia, studies have shown a variation between 59% [18] and 98% [22] of vasogenic oedema depending on selection of participants. Prospective studies show an incidence of silent cerebral infarcts in a third of women with eclampsia [24, 25, 26, 27]. In preeclampsia, cerebral infarcts seem uncommon. In severe disease where cerebral oedema is present, silent cerebral infarcts have been found in 5%–10% of cases [17, 27].

Most cases of eclampsia occur in low‐ and middle‐income countries where access to imaging, such as MRI, may be limited. It would be useful to have a clinical tool able to detect women at highest risk for persisting sequelae. Women at greatest risk for cerebral infarcts could possibly be triaged for priority imaging and extended neurological follow up.

The aim of this study was to explore clinical markers to detect those at highest risk of silent cerebral infarcts among women with eclampsia.

## 2 Materials and Methods

### 2.1 Study Design

Prospective observational study with cross‐sectional MRI assessment.

### 2.2 Setting

Tygerberg Hospital in Cape Town, South Africa, is the largest referral hospital in the Western Cape Province, with over 8500 high‐risk pregnancy deliveries a year, including many women with preeclampsia [28].

### 2.3 Participants

Women with singleton pregnancies recruited to the Preeclampsia Obstetric Adverse Events (PROVE) biobank at Tygerberg Hospital were included [28]. This database and biobank captures a majority of women presenting with preeclampsia at the centre. Women with known neurological or cardiac diseases, or a clinical indication for brain imaging were not eligible. Eligible women underwent brain magnetic resonance imaging according to a pre‐defined protocol.

For the full eclampsia population included in the biobank, see original publication, Table S2 [27].

### 2.4 Variables

Preeclampsia was defined according to the American College of Obstetricians and Gynecologists Practice Bulletin but significant proteinuria was also required to diagnose preeclampsia (protein creatinine ratio ≥ 30 mg/mmol, ≥ 0.3 g protein in a 24 h urine collection, or a urine dipstick reading > 1+ on more than one occasion) [29]. Pulmonary oedema was diagnosed when there was worsening dyspnoea, fine bibasal inspiratory crackles on auscultation and features of pulmonary oedema on chest X‐ray or CT scan. Haemolysis, elevated liver enzymes and low platelets (HELLP) syndrome was defined as a platelet count < 100 × 10^9^/L, aspartate aminotransferase > 70 U/L and haemolysis as demonstrated by lactate dehydrogenase > 600 U/L or haemolysis on a peripheral blood smear. Eclampsia was diagnosed if generalised tonic–clonic seizures occurred in a woman diagnosed with preeclampsia in the absence of another aetiology. Recurrent eclampsia was defined as more than one seizure after a diagnosis of eclampsia. Renal impairment was defined as a serum creatinine above 120 μmol/L (highest recorded) which is higher than the American College of Obstetricians and Gynecologists (ACOG) definition. Women were followed from recruitment until hospital discharge. Severe hypertension was defined as a systolic blood pressure ≥ 160 mmHg and/or a diastolic blood pressure ≥ 110 mmHg.

Potential predictors for cerebral infarcts in eclampsia were selected from the PROVE database, which was developed using a standardised framework for preeclampsia research [28, 30]. A standardised questionnaire capturing signs and symptoms associated with eclampsia was established by a transdisciplinary team within the research group. These data were systematically collected for all women at the time of inclusion in the study [28]. The full questionnaire is provided in the Supporting Information.

Blood pressure was measured with a validated device for pregnancy, within the clinical setting and information pulled from the medical charts. At this point, women were not yet included in the study. Highest blood pressure was recorded irrespective of concurrent medications.

### 2.5 Data Sources

Baseline data were obtained by interview and extraction from medical records. All data were entered and stored on a Research Electronic Data Capture database [31] and audited for accuracy.

### 2.6 Outcomes Measures

MRI was performed on a 1.5‐T scanner (Aera, Siemens Healthcare, Erlangen, Germany). A 2D diffusion‐weighted imaging (DWI) sequence was acquired with repetition time/echo time/flip angle: 6500 ms/119 ms/90 degrees, spatial resolution 1.25 × 1.25 × 3 mm and one excitation. Nine *b*‐values (0, 50, 100, 150, 200, 400, 600, 800 and 1000 s/mm^2^ × 10^−3^) were used for separation of diffusion and perfusion parameters, as has been previously reported [27].

Primary image evaluation was performed by a radiologist with five years' experience and subsequently reviewed by a neuroradiologist with 30 years' experience. Cerebral infarcts were defined as high intensity lesions on the *b*_1000_ DWI images with corresponding low intensity on the apparent diffusion coefficient (ADC) map and not compatible with seizure‐related lesions. Infarcts were evaluated with respect to anatomical location, number per woman, size of largest infarct, and ADC value in the infarct with the most prominent ADC decrease. The presence of cerebral infarcts was used as the outcome due to its clinical importance.

### 2.7 Sample Size

Previous studies found around 30% prevalence of cerebral infarcts on MRI in eclampsia [24, 25, 26, 27]. In our population, one third of women with eclampsia had cerebral infarcts (*n* = 16), as previously reported by our group [27]. Although no formal sample size calculation was conducted, this is one of the largest cohorts to date of women with eclampsia and MRI imaging. The present study serves as a pilot study for predictors of cerebral infarcts in preeclampsia and eclampsia.

### 2.8 Statistical Methods

Descriptive data were summarised as mean and standard deviation (SD) or median and interquartile range (IQR) for numeric variables, as appropriate, and as numbers and percentages for categorical variables. Baseline differences between women with and without cerebral infarcts were assessed using absolute standardised mean differences, with larger values indicating greater imbalance between groups.

In univariable analyses, predictors of cerebral infarcts were evaluated using log‐linear Poisson regression to estimate the relative risk of infarcts. Robust standard errors (HC3 method) were applied to account for violations against distributional assumptions. Variables that were significant at the 20% level in univariable analyses were considered for multivariable analyses. Multivariable analyses were conducted using logistic regression, with model selection through stepwise (forward‐backward) selection, using the Akaike Information Criterion (AIC) as the selection criterion. Missing data were generally limited (< 10%), and all analyses were conducted on an available‐case basis.

Model performance was evaluated using receiver operating characteristic (ROC) curve analysis. An optimal risk cut‐off for cerebral infarcts was identified using Youden's *J* index, maximising the sum of sensitivity and specificity. Sensitivity and specificity, as well as positive and negative predictive values, were calculated with corresponding 95% Wilson score confidence intervals. In addition, positive and negative likelihood ratios with 95% confidence intervals were calculated using Simel's method.

Statistical analyses were performed using SAS/STAT Software, Version 9.4 (SAS Institute Inc., Cary, NC, USA) and R Software, version 4.2.3 (R Core Team, Vienna, Austria). Confidence intervals for the positive and negative likelihood ratios were calculated by using the epiR package, version 2.0.63 in R.

### 2.9 Ethics Approval and Registration Details

Ethics approval was obtained from Stellenbosch University Health Research Ethics Committee (protocol number N18/03/034, Federal Wide assurance number 00001372, Institutional Review Board number IRB0005239). All participants or their guardians signed informed consent. The biobank is registered (ISRCTN10623443) and the protocol is published [28].

## 3 Results

### 3.1 Participants

**FIGURE 1:**
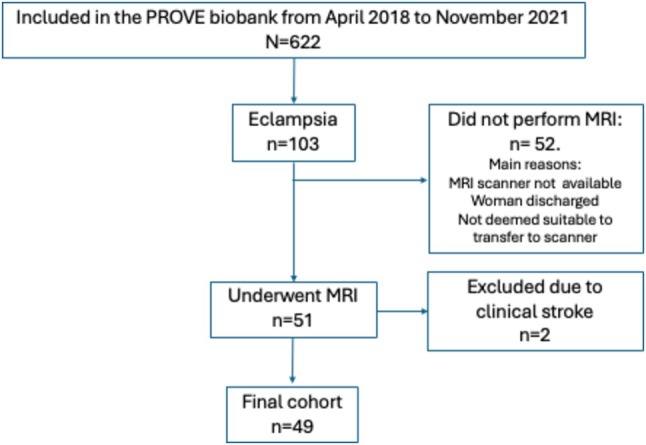
Selection of the study cohort from the PROVE biobank. Of 103 women with eclampsia, 51 underwent brain magnetic resonance imaging. Two were excluded due to clinically evident stroke, resulting in a final cohort of 49 women.

There were 103 women with eclampsia included in the PROVE database from April 2018 to November 2021. Fifty‐one (50%) of these women underwent MRI. The most common reason for not performing MRI was unavailability of the MRI camera at the time of inclusion. Of women who underwent MRI, two were excluded due to clinically evident stroke. Finally, 49 women with eclampsia were included (Figure 1). None had a clinical indication for imaging, and MRI was performed for research purposes only. Median (IQR) time from seizure to MRI was 2 (1–3) days.

### 3.2 Maternal Characteristics and Pregnancy Outcomes

Baseline characteristics are presented in Table 1. The included women had a mean age of 22.4 years (SD 5.6) and a BMI of 24.8 (SD 5.6). Thirty‐six women (74%) were nulliparous and 41 (84%) had attended any antenatal care. Six women (12%) were HIV positive and nine (18%) were current smokers. Forty‐one women (84%) gave birth to a liveborn infant, and the median gestational age at birth was 34 weeks and 4 days.

**TABLE 1:** Baseline characteristic of the study cohort, overall and stratified by presence of cerebral infarction.

Characteristic	Full cohort (*n* = 49)	Without infarction (*n* = 33)	With infarction (*n* = 16)	Standardised mean difference
Maternal demographics				
Age (years), mean (SD)	22.5 (5.7)	22.8 (5.8)	21.7 (5.7)	0.20
BMI (kg/m^2^), mean (SD)	24.6 (5.6)	24.5 (6.1)	25.1 (4.6)	0.11
Nulliparous, *n* (%)	35 (71%)	23 (70%)	12 (75%)	0.12
Any antenatal care, *n* (%)	41 (84%)	28 (85%)	13 (81%)	0.10
HIV positive, *n* (%)	6 (12%)	4 (12%)	2 (13%)	0.01
Smoking during pregnancy, *n* (%)	10 (20%)	8 (24%)	2 (13%)	0.29
Alcohol use during pregnancy, *n* (%)	6 (12%)	4 (12%)	2 (13%)	0.01
Diabetes mellitus, *n* (%)	0 (0%)	0 (0%)	0 (0%)	0.00
Antenatal care and delivery				
Highest sBP before fit (mmHg), mean (SD)	167 (23)	162 (19)	178 (28)	0.72
> 140 mmHg, *n* (%)	45 (92%)	29 (88%)	16 (100%)	
> 160 mmHg, *n* (%)	25 (51%)	15 (45%)	10 (63%)	
> 180 mmHg, *n* (%)	10 (20%)	4 (12%)	6 (38%)	
> 190 mmHg, *n* (%)	8 (16%)	2 (6%)	6 (38%)	
Highest dBP before inclusion/fit (mmHg), mean (SD)	106 (19)	106 (18)	107 (23)	0.08
Impaired hearing, *n* (%)	8 (17%)	3 (10%)	5 (33%)	0.64
Mode of birth, *n* (%)				
Vaginal	18 (37%)	12 (36%)	6 (38%)	0.02
Planned CS	1 (2%)	0 (0%)	1 (6%)	0.46
Emergency CS	30 (61%)	21 (64%)	9 (56%)	0.15
Gestational age at delivery (weeks + days), median (range)	34 + 4 (24 + 2–40 + 5)	35 + 5 (24 + 5–40 + 5)	31 + 3 (24 + 2–40 + 3)	0.48
Gestation at delivery < 34 weeks, *n* (%)	26 (53%)	15 (45%)	11 (69%)	
Gestation at delivery < 37 weeks, *n* (%)	39 (80%)	26 (79%)	13 (81%)	
Maternal outcomes				
Maternal death, *n* (%)	0 (0%)	0 (0%)	0 (0%)	0.00
Intensive care unit admission, *n* (%)	4 (8%)	2 (6%)	2 (13%)	0.23
Recurrent eclampsia, *n* (%)	16 (33%)	11 (33%)	5 (31%)	0.04
Glasgow Coma Scale < 13, *n* (%)	11 (22%)	6 (18%)	5 (31%)	0.31
Cortical blindness, *n* (%)	4 (8%)	4 (12%)	0 (0%)	0.44
Pulmonary oedema, *n* (%)	2 (4%)	2 (6%)	0 (0%)	0.30
Inotropic support, *n* (%)	1 (2%)	0 (0%)	1 (6%)	0.44
Renal impairment, *n* (%)	11 (22%)	8 (24%)	3 (19%)	0.13
Dialysis, *n* (%)	1 (2%)	0 (0%)	1 (6%)	0.44
HELLP syndrome, *n* (%)	13 (27%)	9 (27%)	4 (25%)	0.05
DIC INR > 1.2, *n* (%)	10 (20%)	5 (15%)	5 (31%)	0.41
Severe hypertension, *n* (%)	18 (37%)	9 (27%)	9 (56%)	0.63
Sepsis, *n* (%)	4 (8%)	3 (9%)	1 (6%)	0.10
Venous thromboembolism, *n* (%)	1 (2%)	1 (3%)	0 (0%)	0.21
Placental abruption, *n* (%)	4 (8%)	1 (3%)	3 (19%)	0.58
Neonatal outcomes				
Liveborn infant, *n* (%)	41 (84%)	28 (85%)	13 (81%)	0.10
Birthweight (g), mean (SD)	2070 (930)	2170 (910)	1860 (970)	0.33

*Note:* Continuous variables are presented as mean (standard deviation, SD) or median (range), as appropriate. Categorical variables are presented as count (percentage).

Abbreviations: BMI, body mass index; BP, blood pressure; CS, caesarean section; dBP, diastolic blood pressure; DIC, disseminated intravascular coagulation; HELLP, haemolysis, elevated liver enzymes and low platelet count; HIV, human immunodeficiency virus; ICU, intensive care unit; INR, international normalised ratio; sBP, systolic blood pressure; SD, standard deviation.

Other preeclampsia‐related complications were common. One woman (2%) had pulmonary oedema, 10 (20%) had renal impairment, 14 (29%) had HELLP syndrome, and 17 (35%) had severe hypertension.

Women with cerebral infarcts more often had a systolic blood pressure > 190 mmHg (38% vs. 6%), and the mean systolic blood pressure before eclamptic fit was higher (178 mmHg [SD 29] vs. 162 mmHg [SD 19]) compared with women without cerebral infarcts. Women with cerebral infarcts more often reported acute hearing impairment (33% vs. 10%). Further, these women gave birth at a lower gestational age (median 31 + 3 vs. 35 + 5 weeks + days). Regarding maternal outcomes, women with cerebral infarcts were more frequently admitted to the intensive care unit (13% vs. 6%) and more often had a Glasgow Coma Scale score < 13 (31% vs. 18%). They were also more likely to experience coagulation abnormalities (INR > 1.2; 31% vs. 15%), severe hypertension (systolic blood pressure > 160 mmHg, 56% vs. 27%) and placental abruption (19% vs. 3%) compared with women without cerebral infarcts. Neonatal outcomes were similar between groups. All women were treated with Magnesium sulphate and at least one antihypertensive drug.

### 3.3 Cerebral Infarcts

**FIGURE 2:**
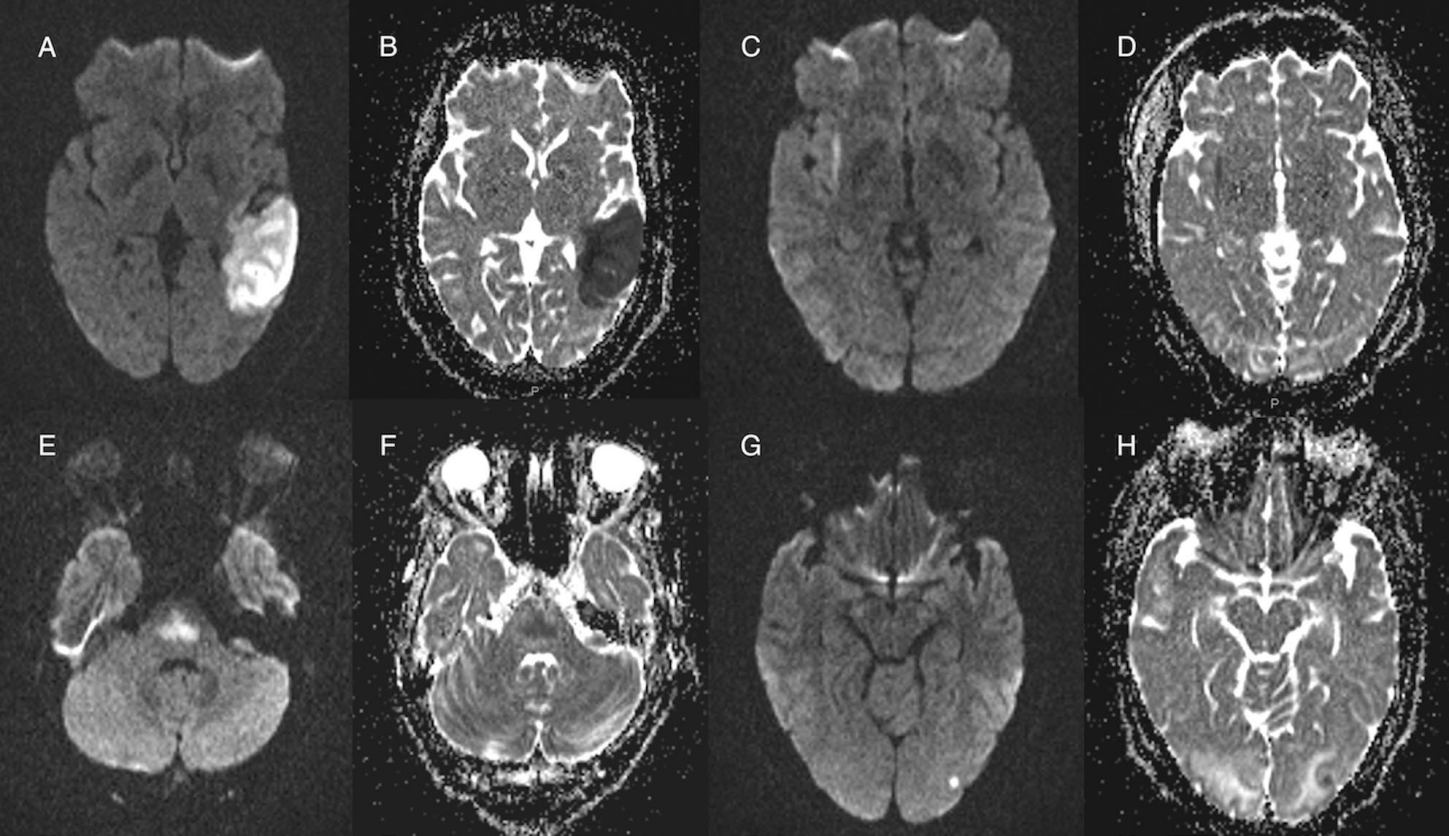
Images of four study participants with infarcts detected on diffusion‐weighted MRI. Images A, C, E and G are from diffusion‐weighted sequences with a *b*‐value of 1000 s/mm^2^. Images B, D, F and H are apparent diffusion coefficient (ADC) maps. Cytotoxic edema in acute infarcts is characterised by high signal intensity on diffusion‐weighted images and low signal intensity in ADC images. Images A and B (participant 1) show a large infarct in the posterior part of the left medial cerebral artery territory. Images C and D (participant 2) show a linear infarct in the right external capsule. Images E and F (participant 3) show an infarct in the pons, and images G and H (participant 4) show a small, rounded infarct in the left occipital cortex. Subcortical high signal in image H corresponds to areas of vasogenic oedema related to posterior reversible encephalopathy syndrome (PRES).

Sixteen women (33%) had cerebral infarcts. The number of infarcts per woman, anatomical location, size and ADC values are presented in Table S1, and representative diffusion‐weighted MRI images are shown in Figure 2.

### 3.4 Clinical Predictors of Cerebral Infarcts

**FIGURE 3:**
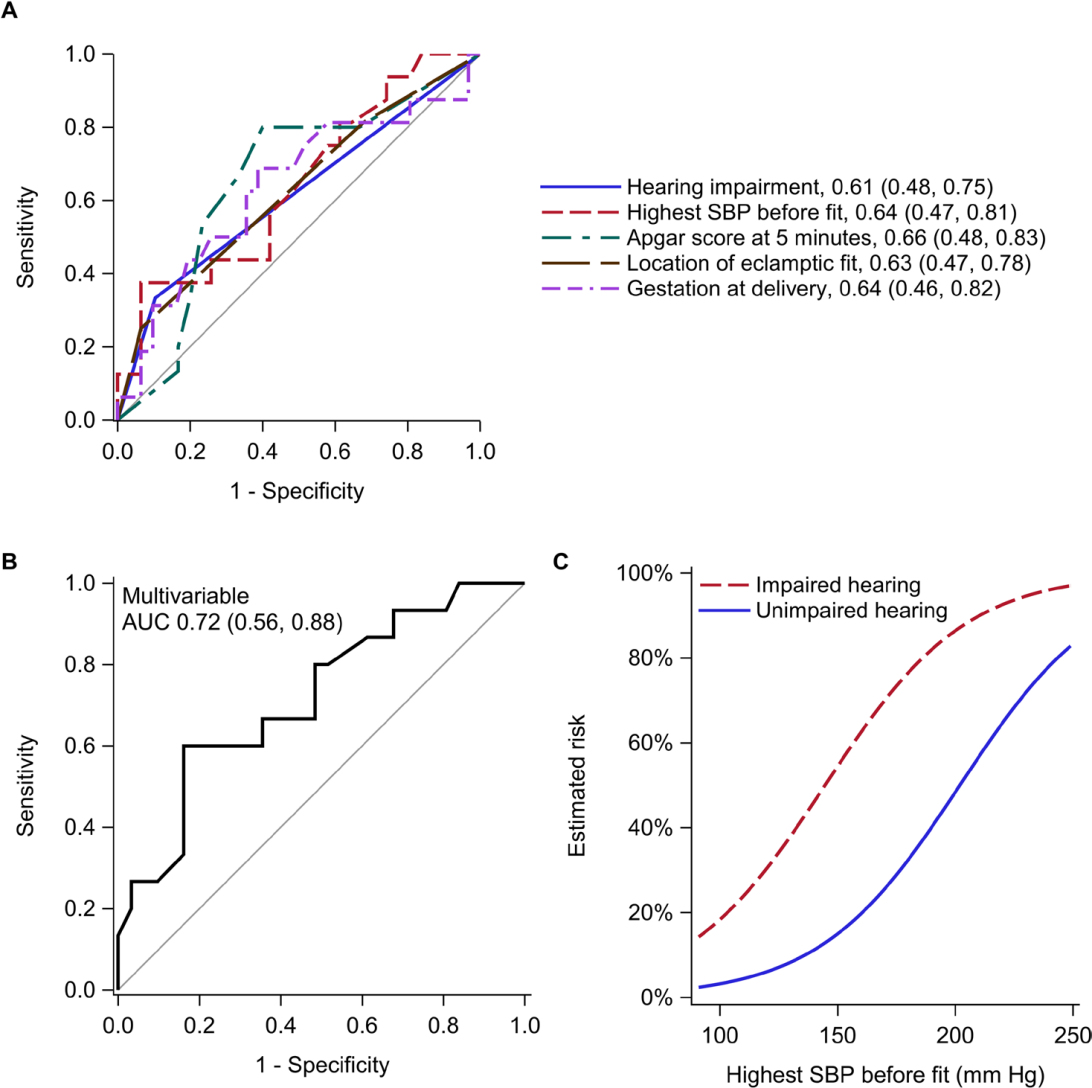
Clinical markers associated with cerebral infarcts in women with eclampsia. (A) Receiver operating characteristic (ROC) curves for candidate predictors for cerebral infarcts significant at the 20% level in univariable analyses. Numbers indicate the area under the ROC curve (AUC) with 95% confidence intervals. (B) ROC curve for the combined analysis including hearing impairment and highest systolic blood pressure. (C) Estimated risk of cerebral infarcts according to hearing impairment and highest systolic blood pressure prior to the eclamptic seizure.

Univariable associations between candidate clinical variables and cerebral infarcts are summarised in Table 2 and Figure 3A for variables with *p* < 0.20; full univariable results, including missing data, are provided in Table S2.

**TABLE 2:** Univariable analyses of candidate predictors for cerebral infarcts in women with eclampsia (*n* = 49); variables with *p* < 0.20 are shown.

Characteristic	Category	Event rate	Risk ratio (95% CI)	*p*
Hearing impairment	No (reference)	10/38 (26.3%)		
	Yes	5/8 (62.5%)	2.37 (1.02, 5.53)	0.045
Highest SBP before inclusion/fit (per 10 mmHg)	< 165 mmHg	6/24 (25.0%)		
	≥ 165 mmHg	10/25 (40.0%)	1.18 (1.05, 1.34)	0.008
Apgar score at 5 min	< 8	10/21 (47.6%)	1.00	
	≥ 8	5/26 (19.2%)	0.92 (0.82, 1.02)	0.12
Location of first eclamptic seizure	In hospital (reference)	3/13 (23.1%)		
	In an MOU or transit	4/6 (66.7%)	2.89 (0.78, 10.7)	0.11
	At home	9/28 (32.1%)	1.39 (0.40, 4.84)	0.59
Gestation at delivery (per week)	< 34 + 4 weeks + days	11/24 (45.8%)		
	≥ 34 + 4 weeks + days	5/25 (20.0%)	0.93 (0.85, 1.03)	0.15

*Note:* Event rates are shown as the number of women with cerebral infarcts divided by the total number in each category (%). Risk ratios (RRs) and 95% confidence intervals were estimated using Poisson regression with a log link and robust standard errors (HC3 method). For continuous variables, event rates are presented for values above versus below the median. Risk ratios correspond to the following unit increases: Apgar score per one‐unit increase; systolic blood pressure per 10 mmHg; and gestation at delivery per one‐week increase.

Abbreviations: Apgar, appearance, pulse, grimace, activity, respiration score; CI, confidence interval; SBP, systolic blood pressure.

Five variables met the predefined *p* < 0.20 threshold and were carried forward for further modelling. These were highest systolic blood pressure (RR 1.18 per 10 mmHg increase, 95% CI 1.05–1.34; *p* = 0.008), hearing impairment (RR 2.37, 95% CI 1.02–5.53; *p* = 0.045), Apgar score at 5 min (RR 0.92 per unit increase, 95% CI 0.82–1.02; *p* = 0.12), location of the first eclamptic seizure (midwife‐led obstetric unit or in transit vs. in hospital; RR 2.89, 95% CI 0.78–10.7; *p* = 0.11), and gestational age at delivery (RR 0.93 per week increase, 95% CI 0.85–1.03; *p* = 0.15).

In total, eight women had hearing impairment. Of these, five had cerebral infarcts. One of these cerebral infarcts was found in the auditory region.

### 3.5 Multivariable Prediction Model for Cerebral Infarcts

The five candidate predictors identified in univariable analyses were subsequently entered into multivariable analyses (Figure 3). After stepwise selection, only highest systolic blood pressure and hearing impairment were retained in the model, with odds ratios of 1.40 (95% CI 1.01–1.93) per 10 mmHg increase in systolic blood pressure and 6.78 (95% CI 1.20–38.2) for impaired versus unimpaired hearing. The final model was:

$$\hat{p}={\left(1+e^{6.7507-1.9140\times \left(\text{hearing impairment}\right)-0.0334\times \left(\text{highest systolic blood pressure}\right)}\right)}^{-1},$$

where $\hat{p}$ is the predicted probability of a cerebral infarct, hearing impairment coded as present or absent, and systolic blood pressure expressed in mmHg. Three participants were excluded from the final model because of missing information on hearing impairment.

The model achieved an area under the receiver operating characteristic curve of 0.72 (95% CI 0.56–0.88) (Figure 3B). An identified risk threshold of 43%, maximising the sensitivity and specificity, corresponded to impaired hearing with SBP ≥ 140 mmHg (*n* = 8) or unimpaired hearing with SBP ≥ 195 mmHg (*n* = 6; Figure 3C). At this threshold, 9 of 15 women with infarct were correctly classified (sensitivity 0.60, 95% CI 0.36–0.80), and 26 of 31 women without infarcts were correctly classified (specificity 0.84, 95% CI 0.67–0.93). The positive predictive value was 0.64 (9/14; 95% CI 0.39–0.89) and the negative predictive value was 0.81 (26/32; 95% CI 0.68–0.95). The positive and negative likelihood ratios were 3.72 (95% CI 1.51–9.18) and 0.48 (95% CI 0.25–0.90), respectively, indicating moderate predictive performance.

## 4 Discussion

### 4.1 Main Findings

In this study of women with eclampsia undergoing brain MRI, higher systolic blood pressure and impaired hearing were the clinical features most strongly associated with cerebral infarcts. When combined, these features showed moderate discriminatory performance, with an area under the receiver operating characteristic curve of 0.72, a sensitivity of 60%, and a specificity of 84% at a 43% risk threshold.

### 4.2 Interpretation

Only one previous study, including 236 women, investigated predictors of cerebral infarcts in preeclampsia and eclampsia [17]. They found visual disturbances to be a risk factor for cerebral infarcts [17]. In our study, a similar trend was seen but this but did not reach statistical significance. These different findings may be due to the retrospective nature of their study, including women who had a clinical indication for MRI where neurological signs and symptoms used in the clinic would be an indication for imaging, introducing selection bias. In addition, their study included both women with preeclampsia and eclampsia and relied on symptoms that were registered in the medical charts. In our cohort, visual disturbances were common, with most women (38/49) reporting visual symptoms. In contrast, the previous study included both women with preeclampsia and eclampsia. As visual symptoms may be more frequent in eclampsia, and cerebral infarcts may also be more common in this group, an observed association with visual disturbances could be driven by differences between diagnostic groups rather than by visual symptoms per se. These reported signs and symptoms rely on the discretion of the clinician in charge. Therefore, this study also did not include alternative signs and symptoms, such as hearing impairment. We systematically collected signs and symptoms prospectively for all included women. Hearing impairment is a novel clinical feature for cerebral complications in this population. As symptoms were collected using a structured questionnaire, detailed information on the nature, onset or duration of the hearing impairment was not available, as responses were recorded only as the presence or absence of symptoms. Future studies could explore whether more detailed characterisation of hearing impairment provides additional specificity for identifying cerebral involvement. The cortical area responsible for auditory processing is located in the temporal lobe. Among women reporting hearing impairment, infarcts involving the temporal region was present in one case. In addition to the direct relation to auditory region, hearing deficit could also be connected to a general inflammatory generated vasospasm, also affecting the inner ear and might associate to a general increased risk of infarcts, independent of location [32]. To our knowledge, there are no previous prospective studies evaluating clinical features associated with cerebral infarcts in women with eclampsia. We examined a broad range of candidate variables, including baseline characteristics, biochemical markers and neurological symptoms. Despite the inclusion of multiple indicators of disease severity and neurological involvement, most variables were not associated with cerebral infarcts. Higher systolic blood pressure and hearing impairment were the clinical features most strongly associated with cerebral infarcts in this prospective cohort of women with eclampsia.

### 4.3 Clinical Implications

Research from India and our group in South Africa both show that eclampsia is associated with irreversible injury in one third of cases [24, 25, 26, 27]. These findings add to the growing body of evidence that the effects of eclampsia are not completely reversible and may cause permanent damage. This supports previous reports of increased risk of neurological long‐term sequelae such as epilepsy, cognitive decline and dementia, following eclampsia [10, 13, 33]. It is therefore clear that women who have experienced eclampsia should have longer follow‐up. Women who have cerebral infarcts may be at even higher risk of developing long‐term neurological sequelae and should be prioritised in future research, with opportunities for early intervention explored. The clinical markers identified in this study may help to inform future approaches to identify women at higher risk of adverse neurological outcomes and guide follow‐up, particularly in settings where access to MRI is limited.

Women with focal neurological signs such as hemiparesis, aphasia, hemianopia, prolonged seizures, atypical headache or persistent visual disturbances should always be referred for neuroimaging to confirm intracerebral lesions.

### 4.4 Research Implications

Our findings require verification in an independent cohort. If replicated, these clinical features could potentially be used to help prioritise women for neuroimaging and closer neurological follow‐up. In addition, future research should focus on the implications of cerebral infarcts for long‐term neurological outcomes.

Lastly, since MRI is resource dependent, it might be a challenge to repeat the study in another LMIC setting. At present, MRI is required to diagnose these small infarcts but in the future, there might be techniques available at lower cost to increase applicability of imaging in the LMIC setting.

### 4.5 Strengths and Limitations

This study has several strengths. Women were prospectively included and underwent MRI without a clinical indication, increasing the generalisability of the findings. In addition, signs and symptoms were prospectively recorded using a standardised protocol, minimising reporting bias.

There are also limitations to our study. Imaging was performed on a 1.5 Tesla MRI scanner, which may have limited the sensitivity for detecting more discrete infarcts compared with a 3 Tesla MRI. The sample size was relatively small, which may have limited the precision of estimates and the ability to detect additional predictors of cerebral infarcts. In addition, a relatively large number of candidate variables were evaluated in relation to a limited number of events, which may have introduced some optimism in the observed associations. Furthermore, women who underwent MRI had to be clinically stable for transport between floors in the hospital, which might have introduced selection bias and reduced generalisability to all women with eclampsia.

## 5 Conclusion

In this study of women with eclampsia undergoing brain MRI, higher systolic blood pressure and impaired hearing were most strongly associated with silent cerebral infarcts. Although discriminatory performance was modest and the findings require external validation, these clinical features may help inform future triage decisions and guide prioritisation of neuroimaging and neurological follow‐up.

## Author Contributions

L.B., C.C. and J.W. conceived the study and wrote the first draft of the paper. H.I. provided statistical support. E.L. and A.M. included the women in the study and collected the data. R.P. and S.G.‐R. conducted the MRI investigations. J.W. interpreted the MRI data. All authors provided input on the design of the study and approved the final version of the manuscript.

## Funding

The study was supported by the Swedish Society of Medicine, Märta Lundqvist foundation, Swedish foundation for international cooperation in research and higher education (STINT), Jane and Dan Olssons foundation, Mercy Perinatal, the Swedish Research Council (VR), Centre for Clinical Research Dalarna, and the Preeclampsia foundation. L.B. is a Wallenberg Scholar, supported by the Wallenberg Centre for Molecular and Translational Medicine (WCMTM). C.C. receives salary support from the Mercy Health Foundation and VR.

## Ethics Statement

Ethics approval was obtained from Stellenbosch University Health Research Ethics Committee (protocol number N18/03/034, Federal Wide assurance number 00001372, Institutional Review Board number IRB0005239). The biobank is registered (ISRCTN10623443).

## Conflicts of Interest

The authors declare no conflicts of interest.

## Supporting information


**Data S1:** bjo70225‐sup‐0001‐TablesS1‐S2.pdf.

**Data S2:** bjo70225‐sup‐0002‐Supinfo.pdf.

## Data Availability

Due to participant confidentiality and local legislation, the study data cannot be openly shared. De‐identified data underlying the findings may be made available from the corresponding author and principal investigator upon reasonable request and submission of a written proposal, subject to relevant ethical and legal approvals. The statistical code underlying the analyses and results is available from the corresponding author on request.
