# Supplementary material for: Impaired Hearing and Systolic Blood Pressure as Potential Markers of Cerebral Infarction After Eclampsia: A Cross‐Sectional Study
Source: BJOG. 2026 Mar 24;133(8):1679–88. doi: 10.1111/1471-0528.70225 (PMC13254043; doi:10.1111/1471-0528.70225)
Supplement: Supplementary file 1 — Data S1: bjo70225‐sup‐0001‐TablesS1‐S2.pdf. [file BJO-133-1679-s001.pdf]

Supplementary Table 1. Number, anatomical location, size, and apparent diffusion coefficient (ADC) of cerebral infarcts detected on MRI in women with eclampsia.

Number of infarcts	Location	Size of largest lesion (mm)	Apparent diffusion coefficient ($mm^2/s \times 10^{-3}$)
3	Right parietal cortex	2.7	758
1	Left temporoparietooccipital cortex and white matter (posterior MCA territory)	57.9	338
>20	Bilateral parietooccipital cortex, splenium, basal ganglia	18.5	552
1	Right frontal white matter	7.5	521
1	Right parietal and left frontal cortex	21.1	597
15	Bilateral frontal and parietal, right occipital white matter	3.7	563
2	Bilateral frontal white matter	5.9	677
>20	Bilateral parietal and occipital cortex, bilateral external capsule	10.4	
3	Right parietal and occipital cortex, right external capsule	23.6	343
>20	Bilateral frontal, parietal and occipital cortex and white matter	3.5	496
4	Bilateral occipital cortex, splenium	18.1	245
1	Pons	14.8	328
2	Right parietal cortex	7.3	315
1	Pons	20.2	413
5	Right thalamus, pons	4.8	477
>20	Bilateral parietal and occipital cortex	5.2	551

Supplementary Table 2. Univariable analyses of candidate predictors for cerebral infarcts in women with eclampsia (n=49).

Predictor	Missing, n (%)	Category	Event rate	Risk ratio (95% CI)	*P*
Before pregnancy					
Alcohol use during pregnancy	0 (0%)	No (reference)	14/43 (32.6%)		
		Yes	2/6 (33.3%)	1.02 (0.24, 4.43)	0.97
Number of previous pregnancies with preeclampsia	0 (0%)	1 (reference)	11/34 (32.4%)		
		2	4/10 (40.0%)	1.24 (0.45, 3.39)	0.67
		3	1/5 (20.0%)	0.62 (0.06, 6.22)	0.68
HIV status	0 (0%)	Negative (reference)	14/43 (32.6%)		
		Positive	2/6 (33.3%)	1.02 (0.24, 4.43)	0.97
Smoking during pregnancy	0 (0%)	No (reference)	14/39 (35.9%)		
		Yes	2/10 (20.0%)	0.56 (0.13, 2.45)	0.43
Chronic hypertension	1 (2%)	No (reference)	15/45 (33.3%)		
		Yes	1/3 (33.3%)	1.00 (0.08, 12.22)	1.00
Antenatal care attendance	0 (0%)	Before 20 weeks' gestation (reference)	7/24 (29.2%)		
		Only after 20 weeks' gestation	6/17 (35.3%)	1.21 (0.46, 3.19)	0.69
		None	3/8 (37.5%)	1.29 (0.37, 4.46)	0.69
SBP at first antenatal visit (mm Hg)	8 (16%)	<118 mm Hg	7/19 (36.8%)		
		≥118 mm Hg	6/22 (27.3%)	0.82 (0.58, 1.16)	0.26
DBP at first antenatal visit (mm Hg)	8 (16%)	<71 mm Hg	7/20 (35.0%)		
		≥71 mm Hg	6/21 (28.6%)	1.00 (0.68, 1.48)	1.00
Antihypertensive treatment at booking	5 (10%)	No (reference)	12/42 (28.6%)		

Predictor	Missing, n (%)	Category	Event rate	Risk ratio (95% CI)	*P*
		Yes	1/2 (50.0%)	1.75 (0.10, 31.75)	0.70
Body mass index (kg/m^2)	23 (47%)	<23 kg/m^2	4/13 (30.8%)		
		≥23 kg/m^2	3/13 (23.1%)	1.07 (0.38, 2.99)	0.89
Gestation at first presentation for antenatal care (weeks)	6 (12%)	<17+1 weeks + days	6/20 (30.0%)		
		≥17+1 weeks + days	7/23 (30.4%)	0.99 (0.93, 1.06)	0.83
Before inclusion					
Facial oedema	0 (0%)	No (reference)	6/15 (40.0%)		
		Yes	10/34 (29.4%)	0.74 (0.31, 1.77)	0.48
Visual disturbances	0 (0%)	No (reference)	3/11 (27.3%)		
		Yes	13/38 (34.2%)	1.25 (0.38, 4.10)	0.70
Severe headache	2 (4%)	No (reference)	7/19 (36.8%)		
		Yes	9/28 (32.1%)	0.87 (0.37, 2.06)	0.75
Severe epigastric or abdominal pain	2 (4%)	No (reference)	15/44 (34.1%)		
		Yes	1/3 (33.3%)	0.98 (0.08, 11.96)	0.99
Chest tightness	2 (4%)	No (reference)	11/34 (32.4%)		
		Yes	5/13 (38.5%)	1.19 (0.47, 2.99)	0.71
Shortness of breath	2 (4%)	No (reference)	11/35 (31.4%)		
		Yes	5/12 (41.7%)	1.33 (0.53, 3.30)	0.54
Focal neurological deficits	0 (0%)	No (reference)	15/39 (38.5%)		
		Yes	1/10 (10.0%)	0.26 (0.03, 2.26)	0.22
Vomiting	2 (4%)	No (reference)	10/30 (33.3%)		
		Yes	6/17 (35.3%)	1.06 (0.44, 2.57)	0.90

Predictor	Missing, n (%)	Category	Event rate	Risk ratio (95% CI)	*P*
Nausea	2 (4%)	No (reference)	10/27 (37.0%)		
		Yes	6/20 (30.0%)	0.81 (0.33, 1.98)	0.64
Confusion	3 (6%)	No (reference)	8/27 (29.6%)		
		Yes	7/19 (36.8%)	1.24 (0.51, 3.03)	0.62
Twitching or jerking arms or legs	3 (6%)	No (reference)	13/39 (33.3%)		
		Yes	2/7 (28.6%)	0.86 (0.19, 3.77)	0.83
Reduced responsiveness noted by others	3 (6%)	No (reference)	7/17 (41.2%)		
		Yes	8/29 (27.6%)	0.67 (0.28, 1.62)	0.37
Difficulty concentrating	3 (6%)	No (reference)	5/13 (38.5%)		
		Yes	10/33 (30.3%)	0.79 (0.31, 2.02)	0.61
Speech disturbance	3 (6%)	No (reference)	10/35 (28.6%)		
		Yes	5/11 (45.5%)	1.59 (0.63, 3.99)	0.31
Hearing impairment	3 (6%)	No (reference)	10/38 (26.3%)		
		Yes	5/8 (62.5%)	2.37 (1.02, 5.53)	0.045
Anxiety	3 (6%)	No (reference)	10/26 (38.5%)		
		Yes	5/20 (25.0%)	0.65 (0.25, 1.72)	0.38
Feeling of impending doom	3 (6%)	No (reference)	6/15 (40.0%)		
		Yes	9/31 (29.0%)	0.73 (0.30, 1.78)	0.48
Dizziness	3 (6%)	No (reference)	5/13 (38.5%)		
		Yes	10/33 (30.3%)	0.79 (0.31, 2.02)	0.61
Weakness or paralysis	3 (6%)	No (reference)	10/35 (28.6%)		
		Yes	5/11 (45.5%)	1.59 (0.63, 3.99)	0.31

Predictor	Missing, n (%)	Category	Event rate	Risk ratio (95% CI)	*P*
Highest SBP before inclusion/fit (mm Hg)	0 (0%)	<165 mm Hg	6/24 (25.0%)		
		≥165 mm Hg	10/25 (40.0%)	1.18 (1.05, 1.34)	0.008
Highest DBP before inclusion/fit (mm Hg)	0 (0%)	<104 mm Hg	8/24 (33.3%)		
		≥104 mm Hg	8/25 (32.0%)	1.03 (0.80, 1.33)	0.82
Proteinuria (grams/24 hours)	2 (4%)	<5 grams/24 hours	5/16 (31.3%)		
		≥5 grams/24 hours	11/31 (35.5%)	1.15 (0.62, 2.13)	0.64
Lowest platelet count (platelets/mcL)	6 (12%)	<164 platelets/mcL	6/21 (28.6%)		
		≥164 platelets/mcL	7/22 (31.8%)	1.08 (0.69, 1.67)	0.74
Highest AST (U/L)	24 (49%)	<34 U/L	4/12 (33.3%)		
		≥34 U/L	6/13 (46.2%)	1.08 (0.96, 1.22)	0.20
Lowest haemoglobin (g/dL)	4 (8%)	<12 g/dL	6/23 (26.1%)		
		≥12 g/dL	8/22 (36.4%)	0.97 (0.45, 2.08)	0.93
Highest creatinine (µmol/L)	43 (88%)	<113 µmol/L	1/3 (33.3%)		
		≥113 µmol/L	1/3 (33.3%)	1.65 (0.21, 12.91)	0.54
At delivery					
Mode of birth	0 (0%)	Vaginal (reference)	6/18 (33.3%)		
		Planned CS	1/1 (100.0%)	3.00 (0.34, 26.38)	0.31
		Emergency CS	9/30 (30.0%)	0.90 (0.31, 2.60)	0.84
Liveborn infant	0 (0%)	No (reference)	3/8 (37.5%)		
		Yes	13/41 (31.7%)	0.85 (0.27, 2.67)	0.77
Apgar score at 5 minutes	2 (4%)	<8	10/21 (47.6%)		
		≥8	5/26 (19.2%)	0.92 (0.82, 1.02)	0.12

Predictor	Missing, n (%)	Category	Event rate	Risk ratio (95% CI)	*P*
Location of first eclamptic seizure	2 (4%)	In hospital (reference)	3/13 (23.1%)		
		In an MOU or transit	4/6 (66.7%)	2.89 (0.78, 10.68)	0.11
		At home	9/28 (32.1%)	1.39 (0.40, 4.84)	0.59
Gestation at delivery (weeks)	0 (0%)	<34+4 weeks + days	11/24 (45.8%)		
		≥34+4 weeks + days	5/25 (20.0%)	0.93 (0.85, 1.03)	0.15
Preeclampsia-related complications					
Recurrent eclampsia	0 (0%)	No (reference)	11/33 (33.3%)		
		Yes	5/16 (31.3%)	0.94 (0.36, 2.41)	0.89
Severe renal impairment	0 (0%)	No (reference)	13/38 (34.2%)		
		Yes	3/11 (27.3%)	0.80 (0.24, 2.61)	0.70
HELLP syndrome	0 (0%)	No (reference)	12/36 (33.3%)		
		Yes	4/13 (30.8%)	0.92 (0.33, 2.58)	0.88

Event rates are shown as the number of women with cerebral infarcts divided by the total number in each category (%). Risk ratios (RRs) and 95% confidence intervals (CIs) were estimated using Poisson regression with a log link and robust standard errors (HC3 method). For continuous variables, event rates are presented for values below versus above the median for descriptive purposes. Risk ratios correspond to the following unit increases: Apgar score per one-unit increase; systolic and diastolic blood pressure per 10 mm Hg; gestation at first presentation and gestation at delivery per one-week increase; body mass index per 5 kg/m²; and laboratory variables (proteinuria, platelet count, AST, haemoglobin, and creatinine) per 50% increase.

Abbreviations: Apgar, appearance, pulse, grimace, activity, respiration score; AST, aspartate aminotransferase; CI, confidence interval; DBP, diastolic blood pressure; GW, gestational weeks; HELLP, haemolysis, elevated liver enzymes, and low platelet count; HIV, human immunodeficiency virus; MOU, midwife-led obstetric unit; ref, reference; SBP, systolic blood pressure.
