# Supplementary material for: Impaired Hearing and Systolic Blood Pressure as Potential Markers of Cerebral Infarction After Eclampsia: A Cross‐Sectional Study
Source: BJOG. 2026 Mar 24;133(8):1679–88. doi: 10.1111/1471-0528.70225 (PMC13254043; doi:10.1111/1471-0528.70225)
Supplement: Supplementary file 2 — Data S2: bjo70225‐sup‐0002‐Supinfo.pdf. [file BJO-133-1679-s002.pdf]

Form 4 Information Before Inclusion

PROVE

Record ID / Participant study number

____

Symptoms before inclusion

Edema
- ☐ None
- ☐ Hands and feet
- ☐ Face

Visual disturbances
- ☐ No visual disturbances
- ☐ Blurred vision
- ☐ Flickering light
- ☐ Loss of vision
- ☐ Sensitivity to light

Time of onset for visual disturbances
- ◯ Less than 24 hours ago
- ◯ 1-3 days ago
- ◯ More than 3 days ago

Did she have a severe headache despite pain-killers?
- ◯ Yes
- ◯ No

Severe epigastric pain
- ◯ Yes
- ◯ No

Did she have tightness in the chest?
- ◯ Yes
- ◯ No

Was she short of breath?
- ◯ Yes
- ◯ No

Focal neurological deficit
- ☐ None
- ☐ One extremity
- ☐ Two extremities
- ☐ > two extremities
- ☐ Loss of speech
- ☐ Facial right side
- ☐ Facial left side
- ☐ Severe vertigo
- ☐ Other

Signs before inclusion

Highest systolic blood pressure (mmHg)

____

Highest diastolic blood pressure (mmHg)

____

Proteinurea
- ◯ Clear
- ◯ Trace
- ◯ 1+
- ◯ 2+
- ◯ 3+

Treatment before the event

Did she recieve any of the following medication before the event?	☐ None of these medications<br>☐ Methyldopa (Hypotone)<br>☐ Nifedipine 10mg (Adalat short acting)<br>☐ Nifedipine XL (Adalat XL long acting)<br>☐ Labetolol bolus<br>☐ Labetolol infusion<br>☐ Neprosol bolus<br>☐ Neprosol infusion<br>☐ Tridil infusion<br>☐ Epidural
Did she receive Magnesium sulphate before the event?	◯ Yes<br>◯ No
